# A Jungle in There: Bacteria in Belly Buttons are Highly Diverse, but Predictable

**DOI:** 10.1371/journal.pone.0047712

**Published:** 2012-11-07

**Authors:** Jiri Hulcr, Andrew M. Latimer, Jessica B. Henley, Nina R. Rountree, Noah Fierer, Andrea Lucky, Margaret D. Lowman, Robert R. Dunn

**Affiliations:** 1 School of Forest Resources and Conservation, University of Florida, Gainesville, Florida, United States of America; 2 Department of Biology and Keck Center for Behavioral Biology, North Carolina State University, Raleigh, North Carolina, United States of America; 3 Department of Plant Sciences, University of California Davis, California, United States of America; 4 Cooperative Institute for Research in Environmental Sciences, University of Colorado, Boulder, Colorado, United States of America; 5 Department of Ecology and Evolutionary Biology, University of Colorado, Boulder, Colorado, United States of America; 6 Department of Entomology, University of Florida, Gainesville, Florida, United States of America; 7 North Carolina Museum of Natural Sciences, Raleigh, North Carolina, United States of America; Field Museum of Natural History, United States of America

## Abstract

The belly button is one of the habitats closest to us, and yet it remains relatively unexplored. We analyzed bacteria and arachaea from the belly buttons of humans from two different populations sampled within a nation-wide citizen science project. We examined bacterial and archaeal phylotypes present and their diversity using multiplex pyrosequencing of 16S rDNA libraries. We then tested the oligarchy hypothesis borrowed from tropical macroecology, namely that the frequency of phylotypes in one sample of humans predicts its frequency in another independent sample. We also tested the predictions that frequent phylotypes (the oligarchs) tend to be common when present, and tend to be more phylogenetically clustered than rare phylotypes. Once rarefied to four hundred reads per sample, bacterial communities from belly buttons proved to be at least as diverse as communities known from other skin studies (on average 67 bacterial phylotypes per belly button). However, the belly button communities were strongly dominated by a few taxa: only 6 phylotypes occurred on >80% humans. While these frequent bacterial phylotypes (the archaea were all rare) are a tiny part of the total diversity of bacteria in human navels (<0.3% of phylotypes), they constitute a major portion of individual reads (∼1/3), and are predictable among independent samples of humans, in terms of both the occurrence and evolutionary relatedness (more closely related than randomly drawn equal sets of phylotypes). Thus, the hypothesis that “oligarchs” dominate diverse assemblages appears to be supported by human-associated bacteria. Although it remains difficult to predict which species of bacteria might be found on a particular human, predicting which species are most frequent (or rare) seems more straightforward, at least for those species living in belly buttons.

## Introduction

The skin of an average human houses trillions of individual bacteria representing hundreds or even thousands of phylotypes [1]. Most of these phylotypes or “species” (acknowledging that bacterial species definitions are vague [2]), are not yet named [3], and are often difficult to cultivate so are known only based on their nucleotide sequence. Studies have begun to characterize consistent differences in the composition of bacterial taxa across the geography of our bodies, for example, between wet (e.g., armpit) and dry (e.g., forearm) habitats [1], [4]. It is still not clear, however, how the diversity in such habitats is structured and, in particular, what makes some phylotypes common and others rare. Commonness might be the result of historically contingent or even chance processes [5], in which common phylotypes might be expected to differ among individual humans or human populations in unpredictable ways. Alternatively, common phylotypes might be predictably common, because they are from lineages with adaptations that predispose them to predictable success in the environment in which they are common [4], [6], [7].

To test whether the commonness of different phylotypes of bacteria on human bodies is predictable, we compared newly collected samples of skin bacteria and archaea from two independent samples of North Americans. The samples were collected from volunteers in a nation-wide citizen science project, Belly Button Biodiversity (http://www.wildlifeofyourbody.org/), and we assessed the microbial communities on the volunteers using multiplex pyrosequencing of 16S rDNA libraries. We tested two predictions derived from research on common and rare multicellular species, such as tropical trees or freshwater fish. The first two predictions we tested were 1) phylotypes frequent in one human population should be predictably frequent in others and 2) frequent phylotypes should also be abundant when present [7], [8]. We define frequent phylotypes as those that occur on many humans and abundant phylotypes are those that are found many times (which is to say, represented by many 16S rDNA “reads” in the pyrosequencing output) on those humans on which they are found. Theoretically, a phylotype might have a high abundance (many reads when present), even if it is very infrequent. While it has long been known that many communities (be they of fish, bacteria or something else) are composed of a few exceptionally frequent and/or abundant species and many more rare species [9], [10], few studies seem to have compared whether abundant species tend to be the same among regions or, as in our case, populations of hosts.

In the context of rain forest trees, species that are both frequent across samples and abundant when present have been referred to as “oligarchs” [6]. In the context of fish communities, they have been called “core species” (which were then contrasted with “occasional species”) [7]. The concepts of oligarchy or core species are similar to, but more specific than the microbiological concept of a “core microbiome”(note, we will focus on the term oligarchy here, rather than “core species,” to avoid confusion between the ecological term core species and the microbiological term “core microbiome”). The core biome has two relatively separate definitions. In some contexts, the core microbiome is described as a set of genes and metabolic functions that are nearly universal among individuals [11], [12], in many cases relatively independent of which phylotypes are present. In other contexts, the term core microbiome is used in a sense more directly tied to our focus, wherein a core microbiome is described as a set of phylotypes that are nearly universal among samples [13]. The oligarchy concept extends this second definition of the “core microbiome” by statistically considering whether the frequency and abundance of phylotypes is predictable or if samples are taxonomically unpredictable assemblages of strains.

Frequent phylotypes might be those phylotypes with specific adaptations for dispersal among and/or competitive success on human hosts. If success is associated with specific adaptations and those adaptations are difficult to evolve, one might expect frequent species to be phylogenetically clustered [14], [15]. Alternatively, if success is independent of specific adaptations for survival on belly buttons or bodies more generally, success may simply be a function of neutral or stochastic processes [16] or the traits necessary for success may be easily evolved. In this context, we tested a third hypothesis, namely that the most frequently encountered bacterial phylotypes (our putative oligarchs, found on greater than 50% of individuals sampled) tend to be more closely related than would be expected among phylotypes chosen randomly from those we sampled as has been suggested to be the case for rain forest trees [6].

The samples of human skin bacteria in our analyses were collected during two separate citizen-science sampling events in which two separate groups of individuals (35 in the first event, 25 in the second) volunteered to swab their own belly buttons. Citizens participated in this study not only in sampling but also in hypothesis generation (via twitter and online comments) and data visualization and were provided with images of bacterial cultures of their samples (www.wildifeofyourbody.org) and lists of the phylotypes discovered during molecular work. Bacteria are common on all parts of the skin, but the belly button offers several advantages. It is an environment that varies relatively little from person to person, in terms of morphology (compared, for example, to the belly itself). It is removed from daily scrubbing, and has the potential to host a less disturbed bacterial community particularly in contrast to frequently washed and exposed parts of the body such as the hands [4]. And last but not least, sampling from belly buttons has also proven of broad interest to the public, which has aided in drawing attention to discussions of the species with which humans are most intimately associated, a key goal of our broader work (www.yourwildlife.org).

## Materials and Methods

### a) Bacterial Samples

Over the past six months, we have sampled over five hundred volunteers for belly button bacteria. We focus on the first two subsamples (60 individuals in total): a sample from the ScienceOnline meeting of science communicators (January 13–15, 2011, Raleigh, NC, USA), and a sample from the Darwin Day at the Museum of Natural Sciences in Raleigh, NC (February 12, 2011). All participants were provided a written Informed Consent form approved by the North Carolina State University’s Human Research Committee (Approval No. 1987). The University’s Human Research Committee has approved this study. Belly buttons were swabbed with sterile cotton tips that were then immersed in 0.5 ml 10% phosphate saline buffer. Swabbing has previously been determined to be as effective as other sampling methods for sampling of human skin bacteria (7). Samples were kept in −20°C. Genomic DNA was extracted from 50 µL of the sediment of centrifuged samples using the PowerSoil DNA extraction kit (MoBio, Inc.), modified according to Lauber et al. [17]. Amplicons were generated using a combination of the universal bacterial/archaeal primers 515F and 806R [18]. The primer 515F was appended with a TC linker and a Roche 454 B pyrosequencing adapter, and the 806R primer was appended with a 12-bp sample-specific barcode sequence, a CA linker, and a Roche 454 A sequencing adapter. The sample-specific, error-correcting barcode allowed for pooling all amplicons in a single pyrosequencing run. All samples, including no-template controls, were PCR-amplified in triplicate following the protocol described in [18], [19]. Amplicons were cleaned using the UltraClean-htp 96-well PCR Clean-up kit (MoBio). The concentration of each amplicon was determined using the Quant-iT PicoGreen dsDNA kit (Invitrogen), and equimolar aliquots of all samples were pooled. Pyrosequencing was carried out on a Roche Genome Sequencer FLX system running the Titanium chemistry at Engencore (University of South Carolina, USA). The 454 platform was chosen over other platforms, since our team has had significant success with this methodology in previous studies of human microbiome (i.e., [4]). Also, the reads produced by 454 pyrosequencing are significantly longer than from most other approaches, and thus easier to analyze and interpret.

### b) Sequence Data Analyses

The pyrosequencing output consisted of 144,403 reads that passed the first quality screen within the 454 platform. This output was processed and analyzed using the comprehensive analysis package QIIME for barcoded amplicons of microbial communities [20]. All analytical steps described below are part of the QIIME package. The sequencing output was filtered to contain only sequences with length >200 and <1000 bp with an average quality score >25 and no ambiguous characters. Sequences were assigned to samples according to the 12-bp barcode; only 50 sequences had uncorrectable barcode sequence. Sequences that were ≥97% similar were grouped into Operational Taxonomic Units (OTUs) using the uclust method. Representative sequences from all OTUs were aligned with PyNAST [21] according to the RDP template, and the taxonomic identity of each OTU was determined using the RDP Classifier [22] with minimum alignment length 190 and minimum sequence identity 70%. Read counts (a proxy for abundance) of identified microbial taxa across samples were exported as a matrix to be used in subsequent community analyses. All samples were rarefied to a sequencing depth of 400 reads per sample prior to downstream analyses. To confirm that singletons in our dataset are not sequencing artifacts, we performed a manual chimera check of twenty single-copy OTUs unidentified by the RDP classifier in QIIME (chimera suspects) directly against GenBank. None of these twenty sequences was chimeric, thus we concluded that chimeras, even if present, were rare and inconsequential in our dataset.

### c) Analyses

To test whether the frequency and abundance of bacterial phylotypes among humans are predictable, we calculated the Spearman rank correlation between the frequencies of the taxa found in both of two independent sets of individuals. This excludes the very rare taxa, most of which were found in only one individual and thus by definition are negatively correlated between the groups in frequency and abundance. The samples contained 35 and 25 individuals, and 305 taxa were observed in both. We also used Spearman’s rank correlation to quantify the degree to which the most frequent phylotypes (when the two sampling events were pooled) were also the most abundant phylotypes (number of reads) when encountered.

Finally, we examined whether the most frequent bacterial phylotypes (those 23 phylotypes that occurred on >50% of individuals) were phylogentically more closely related than phylotypes within randomly drawn samples. This would be expected if frequent phylotypes tend to be from the narrower subset of lineages with adaptations for life on humans, whereas rare phylotypes tend to be more random samples of environmental bacteria. We measured pairwise phylogenetic diversity among the 23 most widespread belly button denizens (phylotypes present on >50% humans), using Kimura 2-parameter distance [23]. Mean pairwise distance was compared to a distribution of the same measure in a 100 randomly drawn sets of 23 phylotypes from the rest of the phylotypes.

## Results and Discussion

### a) The Basics of Belly Button Biodiversity

In the 60 samples of belly buttons considered here, we found 2368 phylotypes of bacteria based on 144,403 sequence reads, excluding sequences of insufficient quality (see Supporting Information S1). These phylotypes likely correspond to far more than 2368 biological species. Our 3% cut-off is standard in microbial studies, but is conservative so that a given phylotype is likely to include multiple species-level lineages. Also, the overall rarefaction curves for belly button bacterial phylotypes failed to level off, suggesting additional phylotypes would have been encountered were more individuals sampled, or were additional human populations considered (whether from different regions or different genetic backgrounds). Even conservatively considering just the 2368 phylotypes, our diversity of bacterial phylotypes was more than twice as great as the species diversity of, for example, North American birds [24] or ants [25].

The vast majority of phylotypes were both infrequent (encountered on few people) and rare (represented by few reads when present; Table 1). Of 2368 total phylotypes, 2188 were present on less than 10% of individuals sampled (Table 1), and most of those were present on just one individual. Conversely, no phylotypes were present on all individuals sampled and just eight phylotypes were present on more than seventy percent of individuals. These eight phylotypes accounted for nearly half (45%) of the total reads of bacteria in our study.

**Table 1:** Most bacterial phylotypes are only found on very few humans; only a minority is more widespread.

	phylotypes	% human samples
phylotypes present on<50% people	2188	1–10
	97	11–20
	31	21–30
	12	31–40
	17	41–50
23 “oligarchs”, present on>50% people	11	51–60
	4	61–70
	2	71–80
	2	81–90
	4	91–100

From a taxonomic perspective, the frequent, abundant phylotypes encountered were dominated by well-known skin bacteria, specifically Staphylococci, Corynebacteria, and several genera of Actinobacteria (e.g., *Micrococcus*) and Clostridiales (e.g., *Anaerococcus*, *Finegoldia*, *Peptidophilus*), Bacilli, as well, to a lesser extent, Gammaproteobacteria (e.g., Acinetobacter). This composition corresponds to the previously reported composition of the skin microbiome in deep sequencing studies [1], [3], [26]. Interestingly, it is also very similar to the taxa recorded in a culture-based study of skin samples from humans in our same study region of North Carolina [26]. The most common skin bacteria in that study were lineages of *Staphylococcus*, *Micrococcus* (within the group *Actinobacteria* above), *Bacillus* (Bacilli above) and then *Acinetobacter*, *Klebsiella*, *Streptomyces* and *Enterobacter*. The frequently encountered genera in the 1975 culture-based study were all also frequently encountered here with the exception of Streptomyces, and *Enterobacter* which were present in the belly button samples but not common and *Klebsiella* which was absent from our samples.

The quantitative dominance of Corynebacteria in bellybuttons is also in line with a previous report [4]. Of special note are three phylotypes of Archaea, a domain of life often found in extreme environments and not previously reported from human skin [1], [27], multiple phylotypes of which we isolated from two independent samples (see online Supporting Information S1). Two of these three phylotypes were from an individual who self-reported not having showered or bathed for several years.

In order to account for differences in numbers of reads from different belly buttons, we rarefied each belly button sample to 400 reads. Rarefying the data decreased the total number of bacterial phylotypes being considered in our analyses to 1380. For this rarefied dataset, we recovered a median diversity of 67 bacterial phylotypes (per 400 reads) per belly button. The most diverse bacterial sample included 107 phylotypes, and the least diverse included 29 phylotypes. In other words, some belly buttons appear more than three times as diverse as others. Such differences have the potential to influence human health and well-being. Several recent studies suggested that skin bacteria have a beneficial effect on skin immune function [28], [29]. Interestingly, our results suggest that when a high diversity of phylotypes is present on the bellybutton skin, most of those phylotypes are rare, infrequent, phylotypes. Thus, if microbial diversity on habitats like the belly button skin plays a role in allergy, the role may be contingent on the rare, infrequent, phylotypes.

### b) The Frequency of Bacterial Phylotypes is Predictable

**Figure 1:**
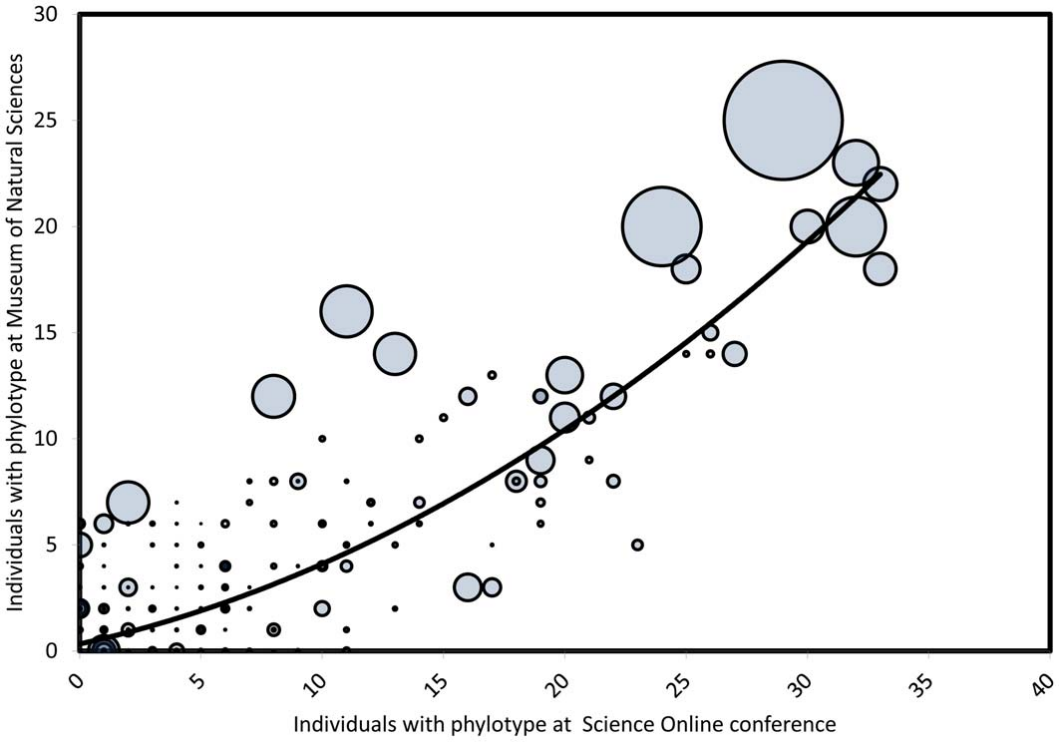
The frequency of bacteria phylotypes (each point  =  a phylotype) in our first sample of human belly buttons predicts most of the variation in the frequency of the same phylotypes in our second sample. The size of circles corresponds to the number of reads of each phylotype, where reads are a proxy for abundance. 1380 points are plotted here, though many fall on top of each other.

While the great diversity of bacterial (and to a far lesser extent, archaeal) phylotypes in belly buttons, like that in many samples of bacteria from humans, suggests an inscrutable complexity, we found that most of the variation in the frequency of phylotypes was predictable. Based on the frequency of bacterial phylotypes (number of hosts on which they occurred) in our first sample of 35 individual humans, we were able to account statistically for much of the variation in the frequency of phylotypes in our second, independent, sample of 25 separate individual humans (Figure 1). Considering the phylotypes that were observed at least once in each sample, frequent phylotypes tend to be predictably frequent and infrequent phylotypes predictably infrequent (Spearman’s ρ = 0.70, P<0.001). Similarly phylotypes abundant in one sample tended to be abundant in the other (ρ = 0.71, P<0.001).

The most frequent bacterial phylotypes tended very strongly to be the most abundant (ρ = 0.90, P<0.001), which is to say a subset of phylotypes is both predictably present and predictably abundant. In short, a subset of bacterial phylotypes, a group we term the oligarchs, are predictably very frequent (despite the great total diversity of bacteria) and often abundant when present.

### c) Frequently Encountered (Oligarchic) Bacteria Phylotypes are Phylogenetically Clustered

**Figure 2:**
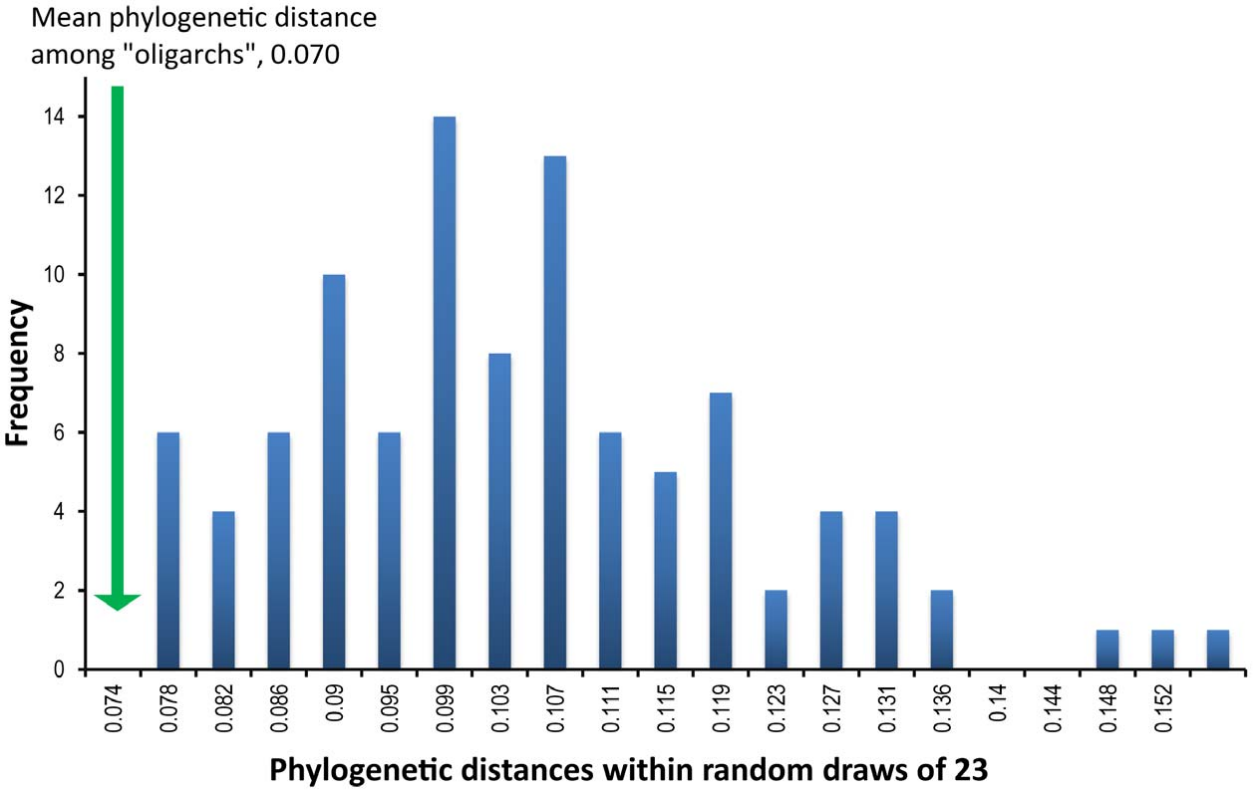
Distribution of pairwise phylogenetic distances among phylotypes. The 23 most frequent phylotypes (which were also, on average, abundant and accounted for 50% of the bacterial reads we encountered) are derived from very few, related, clades (green; 23 phylotypes that occur on >50% of samples; mean pairwise Kimura 2-parameter distance = 0.070), while the remainder of phylotypes were phylogenetically dispersed (blue histogram; distribution of mean pairwise phylogenetic distances among the remaining 2345 phylotypes).

While infrequent, rare phylotypes maybe transient, frequent, and abundant phylotypes (oligarchs) might be expected to be those with specific adaptations to the pH, host antimicrobial compounds and dry conditions that characterize the skin [29]. If this were the case, we would expect the oligarchs to derive from fewer lineages than do more infrequent phylotypes. The hypothesis that frequent taxa are from a subset of lineages with adaptations for the habitat being studied whereas infrequent species draw from a broader range of lineages, many of which are not locally adapted has precedent in ecological literature. For example, a disproportionate number of common rain forest tree species are from the family of palms which possess a range of unique adaptations for tropical forest life [6]. Similarly, only the “core” species of estuarine fish has adaptations for estuary life whereas the many more occasional species are not biologically associated with those habitats [7]. Rare, more occasional, species might represent more random species capable of arriving in a habitat, but not necessarily succeeding. If this were the case, bacteria frequently encountered on humans should belong to fewer lineages than a random draw of the same number of less frequent species. In our samples from belly buttons, we found the most frequently encountered bacterial phylotypes were indeed more phylogenetically clustered than random draws of the same number of representatives from the remaining bacteria. The mean pairwise distance among the most frequent phylotypes (the 23 that occurred on >50% of sampled humans) was 0.070 (Kimura 2-parameter), significantly outside of the 95% range of distances within randomly sampled sets of 23 phylotypes from the rest of the diversity (median = 0.100, lower 95% quantile = 0.078, Figure 2). These results support the hypothesis that while human bodies encounter many thousands of bacterial phylotypes, the most successful phylotypes are from only a few lineages. We hypothesize that these lineages have, over evolutionary history, evolved traits that allow them to thrive on humans, a hypothesis that seems supported by older culture-based studies in which the lineages we found to be most frequent and abundant are nearly identical to those suggested to have specific adaptations for the tough, desert-like conditions found on human skin [26], [30].

### Conclusions

Overall, we found that while belly button bacterial phylotypes were diverse, aspects of this diversity were predictable. The most frequent and abundant phylotypes were similar across independent populations as well as being phylogenetically clustered. In studies of tropical forests, the species found to be both predictably frequent and abundant where present have been termed oligarchs [6], a term we also use here, or “core species” a term used elsewhere in the ecological literature [7].

Such oligarchs were represented by multiple reads in most sampled human individuals, yet not a single one of the oligarchs is present in all samples. This appears in line with the oligarchy concept or ecological core species concept, but at odds with the traditional concept of core microbiome, defined as subset of taxa that are present in all samples [13]. Such phylotypes may have been present on all of the humans in our study but were undetected. However, given that the oligarchs tend to be abundant when present, and they are the least likely phylotypes to be missed our results conform better to the alternative description of the “core microbiome” as stable at the level of genes and metabolic functions, but flexible in its taxonomic composition due to a high functional redundancy among many taxa [11], [12], though even then we note a key distinction. We found that while the microbial communities in human belly buttons may display some degree of flexibility in the taxonomic composition they appear much more predictable than a random assemblage from a functionally redundant metacommunity. Importantly, this pattern of predictable taxonomic composition is borne predominantly by the oligarchs – frequent, abundant, and phylogenetically clustered symbionts, while the rest of the community appears to be much more stochastic. Notably, this means an all-at-once analysis of a bacterial community without regard to differences in predictability among strains may potentially obscure existing taxonomic patterns [31], general both in terms of their predictability from one group of humans to the next but also in terms of their broad correspondence to patterns observed in other taxa, such as fish and tropical trees, at far different spatial scales.

## Supporting Information

Supporting Information S1 **MS Excel workbook with 4 sheets.** Sheet#1: sample grouping: Two groups of samples originating from two collecting events. Sheet #2: DATA list: “Sample” is a belly button sample identifier; “reads/400” is the number of reads of a particular phylotype in a given sample after a rarefaction to 400 reads per sample. Sheet #3: DATA table lists belly button samples on the top row and phylotype identifiers in the first column. Numbers in the table indicate sequencing read numbers. Sheet #4: “New Taxonomy JH” is a list of taxonomic identifiers for our prokaryotic phylotypes. It originated from an automated RDP-based classifier in QIIME, with manual identification of unclear taxa by NCBI BLAST by JH.
(XLS)
